# 2020 update of the WSES guidelines for the management of acute colonic diverticulitis in the emergency setting

**DOI:** 10.1186/s13017-020-00313-4

**Published:** 2020-05-07

**Authors:** Massimo Sartelli, Dieter G. Weber, Yoram Kluger, Luca Ansaloni, Federico Coccolini, Fikri Abu-Zidan, Goran Augustin, Offir Ben-Ishay, Walter L. Biffl, Konstantinos Bouliaris, Rodolfo Catena, Marco Ceresoli, Osvaldo Chiara, Massimo Chiarugi, Raul Coimbra, Francesco Cortese, Yunfeng Cui, Dimitris Damaskos, Gian Luigi de’ Angelis, Samir Delibegovic, Zaza Demetrashvili, Belinda De Simone, Francesco Di Marzo, Salomone Di Saverio, Therese M. Duane, Mario Paulo Faro, Gustavo P. Fraga, George Gkiokas, Carlos Augusto Gomes, Timothy C. Hardcastle, Andreas Hecker, Aleksandar Karamarkovic, Jeffry Kashuk, Vladimir Khokha, Andrew W. Kirkpatrick, Kenneth Y. Y. Kok, Kenji Inaba, Arda Isik, Francesco M. Labricciosa, Rifat Latifi, Ari Leppäniemi, Andrey Litvin, John E. Mazuski, Ronald V. Maier, Sanjay Marwah, Michael McFarlane, Ernest E. Moore, Frederick A. Moore, Ionut Negoi, Leonardo Pagani, Kemal Rasa, Ines Rubio-Perez, Boris Sakakushev, Norio Sato, Gabriele Sganga, Walter Siquini, Antonio Tarasconi, Matti Tolonen, Jan Ulrych, Sannop K. Zachariah, Fausto Catena

**Affiliations:** 1 Department of Surgery, Macerata Hospital, Macerata, Italy; 2 Department of General Surgery, Royal Perth Hospital, The University of Western Australia, Perth, Australia; 3 Department of General Surgery, Division of Surgery, Rambam Health Care Campus, Haifa, Israel; 4 General Surgery Department, Bufalini Hospital Hospital, Cesena, Italy; 5 General, Emergency and Trauma Surgery Department, Pisa University Hospital, Pisa, Italy; 6 Department of Surgery, College of Medicine and Health Sciences, UAE University, Al-Ain, United Arab Emirates; 7 Department of Surgery, University Hospital Centre Zagreb, Zagreb, Croatia; 8 Trauma Surgery Department, Scripps Memorial Hospital, La Jolla, CA USA; 9 Surgical Department and ICU Department, General Hospital of Larissa, Larissa, Greece; 10 Department of Emergency Surgery, Parma Maggiore Hospital, Parma, Italy; 11 Department of General and Emergency Surgery, ASST, San Gerardo Hospital, Monza, Italy; 12 School of Medicine and Surgery, University of Milan-Bicocca, Milan, Italy; 13 General Surgery and Trauma Team, University of Milano, ASST Niguarda Milano, Milan, Italy; 14 Riverside University Health System Medical Center, Loma Linda University School of Medicine, Moreno Valley, CA USA; 15 Emergency Surgery Unit, San Filippo Neri Hospital, Rome, Italy; 16 Department of Surgery, Tianjin Nankai Hospital, Nankai Clinical School of Medicine, Tianjin Medical University, Tianjin, China; 17 Department of Surgery, Royal Infirmary of Edinburgh, Edinburgh, UK; 18 Gastroenterology and Endoscopy Unit, University Hospital of Parma, Parma, Italy; 19 Department of Surgery, University Clinical Center of Tuzla, Tuzla, Bosnia and Herzegovina; 20 Department General Surgery, Kipshidze Central University Hospital, Tbilisi, Georgia; 21 Department of Digestive Surgery, Guastalla Hospital, Reggio Emilia, Italy; 22 General Surgery, Versilia Hospital, UslNordovest, Tuscany, Italy; 23 Colorectal Unit, Addenbrooke’s Hospital, Cambridge University Hospitals NHS Foundation Trust, Cambridge, UK; 24 Envision Healthcare, Dallas, TX USA; 25 Department of General Surgery, Trauma and Emergency Surgery Division, ABC Medical School, Santo André, SP Brazil; 26 Trauma/Acute Care Surgery & Surgical Critical Care, University of Campinas, Campinas, Brazil; 27 Second Department of Surgery, Aretaieion University Hospital, National and Kapodistrian University of Athens, Athens, Greece; 28 Department of Surgery, Hospital Universitário Terezinha de Jesus, Faculdade de Ciências Médicas e da Saúde de Juiz de Fora, Juiz de Fora, Brazil; 29 Trauma Service, Inkosi Albert Luthuli Central Hospital and Department of Surgery, Nelson R Mandela School of Clinical Medicine, Durban, South Africa; 30 Department of General and Thoracic Surgery, University Hospital Giessen, Giessen, Germany; 31 Clinic for Emergency Surgery, Medical Faculty, University of Belgrade, Belgrade, Serbia; 32 Department of Surgery, Assia Medical Group, Tel Aviv University Sackler School of Medicine, Tel Aviv, Israel; 33 Department of Emergency Surgery, City Hospital, Mozyr, Belarus; 34 General, Acute Care, Abdominal Wall Reconstruction, and Trauma Surgery, Foothills Medical Centre, Calgary, AB Canada; 35 Department of Surgery, The Brunei Cancer Centre, Jerudong Park, Brunei; 36 Division of Acute Care Surgery and Surgical Critical Care, Department of Surgery, Los Angeles County and University of Southern California Medical Center, University of Southern California, Los Angeles, CA USA; 37 Department of General Surgery, Faculty of Medicine, Erzincan University, Erzincan, Turkey; 38 Global Alliance for Infections in Surgery, Porto, Portugal; 39 Department of Surgery, Westchester Medical Center, New York Medical College, Valhalla, NY USA; 40 Abdominal Center, University Hospital Meilahti, Helsinki, Finland; 41 Surgical Disciplines, Immanuel Kant Baltic Federal University/Regional Clinical Hospital, Kaliningrad, Russian Federation; 42 Department of Surgery, School of Medicine, Washington University, Saint Louis, USA; 43 Department of Surgery, University of Washington, Seattle, WA USA; 44 Department of Surgery, Post-Graduate Institute of Medical Sciences, Rohtak, India; 45 Department of Surgery, Radiology, University Hospital of the West Indies, Kingston, Jamaica; 46 Department of Surgery, Denver Health Medical Center, University of Colorado, Denver, CO USA; 47 Department of Surgery, Division of Acute Care Surgery, and Center for Sepsis and Critical Illness Research, University of Florida College of Medicine, Gainesville, FL USA; 48 Department of Surgery, Emergency Hospital of Bucharest, Bucharest, Romania; 49 Infectious Diseases Unit, Bolzano Central Hospital, Bolzano, Italy; 50 Department of Surgery, Anadolu Medical Center, Kocaali, Turkey; 51 General Surgery Department, Colorectal Surgery Unit, La Paz University Hospital, Madrid, Spain; 52 General Surgery Department, Medical University, University Hospital St George, Plovdiv, Bulgaria; 53 Department of Aeromedical Services for Emergency and Trauma Care, Ehime University Graduate School of Medicine, Ehime, Japan; 54 Emergency Surgery, Fondazione Policlinico Universitario A. Gemelli IRCCS, Università Cattolica del Sacro Cuore, Rome, Italy; 55 First Department of Surgery, First Faculty of Medicine, Charles University in Prague and General University Hospital in Prague, Prague, Czech Republic; 56 Department of Surgery, MOSC Medical College Kolenchery, Cochin, India

**Keywords:** Acute left-sided colonic diverticulitis, Acute right-sided colonic diverticulitis, Peritonitis, Abscess

## Abstract

Acute colonic diverticulitis is one of the most common clinical conditions encountered by surgeons in the acute setting. An international multidisciplinary panel of experts from the World Society of Emergency Surgery (WSES) updated its guidelines for management of acute left-sided colonic diverticulitis (ALCD) according to the most recent available literature. The update includes recent changes introduced in the management of ALCD. The new update has been further integrated with advances in acute right-sided colonic diverticulitis (ARCD) that is more common than ALCD in select regions of the world.

## Introduction

Acute left-sided colonic diverticulosis is common in Western countries with its prevalence increasing throughout the world, which is likely due to changes in lifestyle [1]. Although left-sided colonic diverticulosis remains more common among elderly patients, a dramatic rise of its incidence has been seen in younger age groups in recent years [2]. Recent evidence suggests that lifetime risk of developing acute left-sided colonic diverticulitis (ALCD) is about 4% among patients with diverticulosis [3], and data from Western populations suggest that up to one fifth of patients with acute diverticulitis are under 50 years of age [4–6].

ALCD is a common problem encountered by Western surgeons in the acute setting. The sigmoid colon is usually the most commonly involved part, while acute right-sided diverticulitis (ARCD) is rarer but much more common in non-Western populations.

## Methods

The World Society of Emergency Surgery (WSES) guidelines for management of ALCD were published in 2016 [7]. In 2020, the guidelines were revised and updated.

The present guidelines have been developed according to the GRADE methodology [8, 9]. The GRADE system is a hierarchical, evidence-based tool, which systematically evaluates the available literature and focuses on the level of evidence based upon the types of studies included. The quality of evidence can be marked as high, moderate, low, or very low. This could be either downgraded in case of significant bias or upgraded when multiple high-quality studies showed consistent results. The highest quality of evidence studies (systematic reviews with meta-analysis of randomized controlled trials) was assessed first. If the meta-analysis was of sufficient quality, it was used to answer the research question. If no meta-analysis of sufficient quality was found, randomized controlled trials (RCTs) and non-randomized cohort studies (n-RCS) were evaluated. The strength of the recommendation was based on the level of evidence and qualified as weak or strong.

A multidisciplinary panel of experts, coordinated by a central coordinator, was selected to serve as experts in this 2020 update of the WSES guidelines for the management of acute left-sided colonic diverticulitis (ALCD). The experts reviewed and updated the original list of key questions on the diagnosis and treatment of ALCD addressed in the previous version of the guidelines.

For each statement, a consensus among the panel of experts was reached using a Delphi approach. Statements were approved with an agreement of ≥ 80%.

After the approval of the statements, the expert panel met via email to prepare and revise the definitive guidelines. The manuscript was successively reviewed by all members and ultimately revised as the present manuscript.

## Which classification should be used in patients with ALCD?

*There are multiple classification systems for ALCD. None has been conclusively proven to be superior in predicting patient outcomes, and therefore, a specific recommendation cannot be provided.*

ALCD ranges in severity from uncomplicated phlegmonous diverticulitis to complicated diverticulitis including abscess and/or perforation.

For the past three decades, the Hinchey classification has been the most used classification in the international literature [10].

In patients with surgical findings of abscesses and peritonitis, Hinchey et al. classified the severity of acute diverticulitis into four levels:

1. Pericolic abscess
2. Pelvic, intra-abdominal, or retroperitoneal abscess
3. Generalized purulent peritonitis
4. Generalized fecal peritonitis

In recent years, the management of ALCD has changed dramatically.

Computer tomography (CT) imaging has become a primary diagnostic tool in the diagnosis and staging of patients with ALCD, and more detailed information provided by CT scans led to several modifications of the Hinchey classification [4, 11–15].

In 1989, Neff et al. presented a new classification of ALCD based on CT findings. It consisted of five stages, ranging from radiological diagnosis of uncomplicated (stage 0) to pneumoperitoneum with abundant free liquid (stage 4) [11]:

0 Uncomplicated diverticulitis; diverticula, thickening of the wall, increased density of the pericolic fat
1 Locally complicated with local abscess
2 Complicated with pelvic abscess
3 Complicated with distant abscess
4 Complicated with other distant complications

In 2002, Ambrosetti et al. [12] classified ALCD into severe or moderate disease. In this classification, the CT scan determined the grade of severity guiding the physician in the treatment of acute complications. Moderate diverticulitis was defined by wall thickening of ≥ 5 mm and signs of pericolic fat inflammation. Severe diverticulitis was defined by wall thickening accompanied by abscess, extraluminal gas, or extraluminal contrast:

1. Moderate diverticulitis
  - Localized sigmoid wall thickening (≥ 5 mm)
  - Pericolic fat stranding

2. Severe diverticulitis
  - Abscess
  - Extraluminal gas
  - Extraluminal contrast

In 2005, Kaiser et al. [13] modified the Hinchey classification according to specific CT findings:

Stage 0: mild clinical diverticulitis

Stage 1a: confined pericolic inflammation

Stage 1b: confined pericolic abscess

Stage 2: pelvic or distant intra-abdominal abscess

Stage 3: generalized purulent peritonitis

Stage 4: fecal peritonitis at presentation

In 2013, Mora Lopez et al. proposed [14] a modification of the previous Neff classification dividing Neff stage 1 into stage 1a (localized pneumoperitoneum in the form of gas bubbles) and 1b (abscess < 4 cm).

0Uncomplicated diverticulitis. Diverticula, thickening of the wall, increased density of the pericolic fat

1Locally complicated diverticulitis

1aLocalized pneumoperitoneum in the form of gas bubbles

1bAbscess (< 4 cm)

2Complicated diverticulitis with pelvic abscess. Abscess > 4 cm in pelvis

3Complicated diverticulitis with distant abscess. Abscess in abdominal cavity (outside pelvis)

4Complicated diverticulitis with other distant complications. Abundant pneumoperitoneum and/or intra-abdominal free liquid

Recently, Sallinen et al. [15] published an interesting retrospective study of patients treated for ALCD, setting the stage for the treatment of acute diverticulitis based on clinical, radiologic, and physiologic parameters:

1Uncomplicated diverticulitis

2Complicated diverticulitis with small abscess (< 6 cm)

3Complicated diverticulitis with large abscess (≥ 6 cm) or distant intraperitoneal or retroperitoneal gas

4Generalized peritonitis without organ dysfunction

5Generalized peritonitis with organ dysfunction

Finally, a proposal for a CT-guided classification of ALCD was published in 2015 by the WSES acute diverticulitis working group [7].

It is a simple classification system of ALCD based on CT scan findings. It may guide clinicians in the management of acute diverticulitis and may be universally accepted for day to day practice. The WSES classification divides acute diverticulitis into 2 groups: uncomplicated and complicated.

In the event of uncomplicated acute diverticulitis, the infection only involves the colon and does not extend to the peritoneum. In the event of complicated acute diverticulitis, the infectious process proceeds beyond the colon. Complicated acute diverticulitis is divided into 4 stages, based on the extension of the infectious process:

*Uncomplicated*

0Diverticula, thickening of the wall, increased density of the pericolic fat

*Complicated*

1APericolic air bubbles or small amount of pericolic fluid without abscess (within 5 cm from inflammed bowel segment)

1BAbscess ≤ 4 cm

2AAbscess > 4 cm

2BDistant gas (> 5 cm from inflammed bowel segment)

3Diffuse fluid without distant free gas

4Diffuse fluid with distant free gas

## What is the best way to make a diagnosis of ALCD?

*In patients with suspected ALCD, we suggest a complete assessment of the patients using clinical history, signs, laboratorial inflammation markers, and radiological findings (weak recommendation based on very low-quality evidence, 2D).*

*In patients with suspected, ALCD we suggest against diagnosis based only on clinical examination (weak recommendation based on very low-quality evidence, 2D).*

Clinical findings of patients having ALCD include acute pain or tenderness in the left lower quadrant that may be associated with increased inflammatory markers, including C-reactive protein (CRP) and white blood cell count (WBC). Clinical diagnosis of ALCD usually lacks accuracy. In a prospective analysis [16] conducted on 802 consecutive patients who presented with abdominal pain to the emergency department, positive and negative predictive values of clinical diagnosis were 0.65 and 0.98, respectively. Additional cross-sectional imaging had a positive and negative predictive value of 0.95 and 0.99, respectively. Additional radiology examinations improved the diagnostic accuracy in 37% of the patients, but changed the management in only 7%.

In 2010, using logistic regression analysis, Laméris et al. [17] developed a clinical decision rule for diagnosis of ALCD, based on 3 criteria: (1) direct tenderness only in the left lower quadrant, (2) CRP > 50 mg/l, and (3) absence of vomiting. Of 126 clinically suspected patients enrolled in this prospective study, 30 patients had all 3 features (24%), of whom 29 had a final diagnosis of acute diverticulitis (97%; 95% CI 83–99%). Of the 96 patients without all 3 features, 45 (47%) did not have diverticulitis. In a quarter of patients with suspected diverticulitis, the diagnosis could be made clinically based on the combination of the three criteria.

Andeweg et al. in 2011 [18], using retrospective data from 287 patients, developed a clinical scoring system for the diagnosis of ALCD with diagnostic accuracy of 86%. It was based on independent predictors of ALCD including age, a clinical history of one or more previous episodes, localization of symptoms in the lower left abdomen, aggravation of pain on movement, the absence of vomiting, localization of abdominal tenderness on examination in the lower left abdomen, and C-reactive protein of 50 mg/l or higher.

CRP has been identified as a useful biomarker of inflammation, and it may be useful in the prediction of the clinical severity of acute diverticulitis as demonstrated by several recent studies [19–21]. To investigate the value of CRP and of other laboratory parameters of the patients in the prediction of the clinical severity of acute diverticulitis, a retrospective study was published in 2014 [19]. A CRP cutoff value of 170 mg/l significantly discriminated severe from mild diverticulitis (87.5% sensitivity, 91.1% specificity, area under the curve 0.942, *p <* 0.00001). The authors concluded that CRP is a useful tool in the prediction of the clinical severity of acute diverticulitis. A mild episode is very likely in patients with CRP less than 170 mg/l. Those with higher CRP values have a greater probability of undergoing surgery or percutaneous drainage.

In another study, the diagnostic value of serological infection markers and body temperature, in discriminating complicated from uncomplicated diverticulitis, was assessed [20]. A total of 426 patients were included in this study of which 364 (85%) presented with uncomplicated and 62 (15%) with complicated diverticulitis. Only CRP was of sufficient diagnostic value (area under the curve 0.715). The median CRP in patients with complicated diverticulitis was significantly higher than in patients with uncomplicated disease (224 mg/l, range 99–284, vs. 87 mg/l, range 48–151, respectively). Patients with a CRP of 25 mg/l had a 15% chance of having complicated diverticulitis. This increased from 23% at a CRP value of 100 mg/l to 47% for 250 mg/l or higher. The optimal threshold was reached at 175 mg/l with a positive predictive value of 36%, negative predictive value of 92%, sensitivity of 61%, and specificity of 82%.

Mäkelä et al. [21] published a study comparing the CRP values of 350 patients who presented for the first time with symptoms of acute diverticulitis with the CT findings and clinical parameters by means of both univariate and multivariate analyses. CRP cutoff value of 149.5 mg/l significantly discriminated acute uncomplicated diverticulitis from complicated diverticulitis (specificity 65%, sensitivity 85%, area under the curve 0.811, *p =* 0.0001). In multivariate analysis, a CRP value over 150 mg/l and old age were independent risk factors for acute complicated diverticulitis. The mean CRP value was significantly higher in the patients who died (mean CRP of 207 mg/l) than in those who survived (mean CRP of 139 mg/l). In addition, a CRP value over 150 mg/l and free abdominal fluid in CT were independent variables predicting postoperative mortality. The study confirmed that CRP is useful for predicting the severity of acute diverticulitis on admission. The authors concluded that patients with a CRP value higher than 150 mg/l have an increased risk of complicated diverticulitis and should always undergo a CT examination.

In 2018, a prospective study of patients with ALCD was published [22]. All patients underwent CT. Index parameters obtained at the initial evaluation in the emergency unit were analyzed to assess the association with the outcome. Ninety-nine patients were analyzed. Eighty-eight had mild radiological grading (Hinchey Ia/Ib), and 11 had severe radiological grading (Hinchey > Ib) (median index CRP 80 mg/l vs. 236 mg/l, *p <* 0.001). The median CRP level for Hinchey III/IV was 258.5 mg/l (201–297 mg/l). WBC, neutrophils/lymphocytes, serum creatinine, serum glucose, generalized peritonitis, generalized abdominal tenderness, urinary symptoms, and index CRP were related to severe disease. Index CRP was the only independent predictor for Hinchey > Ib (*p =* 0.038). The optimal cutoff value calculated by receiver operating characteristic curve analysis was found to be 173 mg/l (sensitivity 90.9%, specificity 90.9%, *p <* 0.001). All patients who underwent radiologic-guided percutaneous or surgery had an index CRP > 173 mg/l and Hinchey > Ib. However, the authors concluded that CRP should not be used as a predictor of severity if there are concomitant conditions that may affect its baseline levels.

The expert panel states that in very acutely onset disease, CRP values might not have rised yet, since there is a delay of 6–8 h from the onset of the disease, reaching peak at 48 h. Therefore, caution should be used in using low CRP as excluding out acute diverticulitis [23].

## What is the best imaging technique in patients with suspected ALCD? What is the role of ultrasound (US) in patients with ALCD?

*In patients with suspected ALCD, we suggest contrast-enhanced CT scan of the abdomen as the imaging technique of first choice (weak recommendation based on moderate-quality evidence, 2B).*

*We suggest to use US in the initial evaluation of patients with suspected ALCD where it is performed by an expert operator. It has wide availability and easy accessibility. A step-up approach with CT performed after an inconclusive or negative US may be a safe approach for patients suspected of acute diverticulitis (weak recommendation based on moderate-quality evidence, 2B).*

Radiological imaging techniques that are used for diagnosing ALCD in the emergency setting are US and CT. Currently, CT is the established method of choice when compared to US and most guidelines cite the high accuracy and other advantages of CT. This approach is the gold standard for both the diagnosis and the staging of patients with ALCD due to its excellent sensitivity and specificity [24–26]. CT scan can also rule out other diagnoses such as ovarian pathology, or leaking aortic or iliac aneurysm.

CT findings in patients with ALCD may include diverticulosis with associated colon wall thickening, fat stranding, phlegmon, extraluminal gas, abscess formation, or intra-abdominal free fluid. CT imaging can go beyond accurate diagnosis of ALCD. CT criteria may also be used to determine the grade of severity and may drive treatment planning of patients [27]. US is a real-time dynamic examination with wide availability and easy accessibility [28]. Its limitations include operator dependency, poor assessment in obese patients, and difficulty in the detection of free gas and deeply located abscesses [29].

A systematic review and meta-analysis of studies [30] that reported diagnostic accuracy of the clinical diagnosis and diagnostic modalities in patients with suspected diverticulitis was published in 2014. Summary sensitivity estimates for US were 90% (95% CI 76–98%) versus 95% (95% CI 91–97%) for CT (*p =* 0.86). Summary specificity estimates for US were 90% (95% CI 86–94%) versus 96% (95% CI 90–100%) for CT (*p =* 0.04).

Although CT is the most sensitive imaging investigation for patients with suspected acute diverticulitis, a step-up approach with CT performed after an inconclusive or negative US has been proposed as safe and alternative approach for patients with suspected acute diverticulitis [30, 31].

Magnetic resonance imaging, which is not constrained by the operator dependency limitation of compared to US [32, 33], until now is currently difficult to perform at in the emergency department.

## Are immunocompromised patients with ALCD at risk for failure of standard, non-operative treatment?

*We suggest that immunocompromised patients with ALCD be considered at high risk for failure of standard, non-operative treatment (weak recommendation based on very low-quality evidence, 2D).*

Immunocompromised patients are at increased risk for complicated ALCD [34–37]. Immunocompromised patients may fail standard, non-operative treatment. As such, most of these patients require urgent surgical intervention, and this is associated with a significantly higher mortality rate [38].

A recent study by Biondo et al. [39] analyzed the relationship between the different causes of immunosuppression (IMS) and ALCD. Immunocompromised patients were divided in 5 groups according to the causes of IMS: group I, chronic corticosteroid therapy; group II, transplant patients; group III, malignant neoplasm disease; group IV, chronic renal failure; and group V, other immunosuppressant treatments. The rate of emergency surgery was high (39.3%), and it was needed more frequently in group I (chronic corticosteroid therapy). In this study, postoperative mortality was of 31.6% and recurrence rate after successful non-operative management occurred in 30 patients (27.8%).

## Should antibiotics be recommended in immunocompetent patients with uncomplicated acute diverticulitis?

*In immunocompetent patients with uncomplicated diverticulitis without signs of systemic inflammation, we recommend to not prescribe antibiotic therapy (strong recommendation based on high-quality evidence, 1A).*

*In patients requiring antibiotic therapy, we recommend oral administration whenever possible, primarily, because an early switch from intravenous to oral therapy may facilitate a shorter inpatient length of stay (strong recommendation based on moderate-quality evidence, 1B).*

The definition of uncomplicated acute diverticulitis is often vague and poorly defined. Uncomplicated acute diverticulitis is defined as localized diverticular inflammation without any abscess or perforation. A universally accepted classification divides intra-abdominal infections (IAIs) into complicated and uncomplicated [40]. In uncomplicated IAIs, the infection only involves a single organ and does not extend to the peritoneum, while in complicated IAIs, the infectious process extends beyond the organ, causing either localized or diffuse peritonitis [40]. For a better definition of acute diverticulitis in these guidelines, we use the term complicated and uncomplicated according to the classification of IAIs.

Uncomplicated acute diverticulitis is an anatomically confined inflammatory process. CT findings include diverticula, thickening of the wall, and increased density of the pericolic fat. Patients with uncomplicated diverticulitis usually have an indolent course with a low incidence of subsequent complications.

The utility of antibiotics in acute uncomplicated acute diverticulitis has been a point of controversy. In recent years, several studies demonstrated that antimicrobial treatment was not superior to withholding antibiotic therapy, in terms of clinical resolution, in patients with mild unperforated diverticulitis [41]. The current consensus is that uncomplicated acute diverticulitis may be a self-limiting condition in which local host defenses can manage the inflammation without antibiotics in immunocompetent patients. In this context, antibiotics are not necessary in the treatment of uncomplicated disease.

A multicenter randomized trial was published in 2012 by Chabok et al. involving ten surgical departments in Sweden and one in Iceland recruiting 623 patients with computed tomography-confirmed acute uncomplicated left-sided diverticulitis [42]. Patients were randomized to treatment with (314 patients) or without (309 patients) antibiotics. Antibiotic treatment for acute uncomplicated diverticulitis neither accelerated recovery nor prevented complications or recurrence. Therefore, antibiotics should be reserved for the treatment of complicated diverticulitis.

A prospective, single-arm, study overviewed [43] the safety and efficacy of symptomatic (non-antibiotic) treatment for CT-proven uncomplicated acute diverticulitis during a 30-day follow-up period. Overall, 161 patients were included in the study, and 153 (95%) completed the 30-day follow-up. A total of 14 (9%) patients had pericolic gas. Altogether, 140 (87%) patients were treated as outpatients, and 4 (3%) of them were admitted to the hospital during the follow-up. The primary outcome measure of the study was to find the incidence of complicated diverticulitis. None of the patients developed complicated diverticulitis or required surgery, but 2 days (median) after inclusion, antibiotics were given to 14 (9%, 6 orally, 8 intravenously) patients. A recent Dutch randomized controlled trail of observational versus systemic antibiotic treatment (DIABOLO trial) [43] for a first episode of CT-proven ALCD Hinchey stages 1a and 1b confirmed that observational treatment without antibiotics did not prolong recovery and could be considered appropriate in these patients.

This study included 22 clinical sites involving 528 patients; Hinchey modified stages 1a (confined pericolic inflammation or phlegmon) to 1b (pericolic or mesocolic abscess) and Ambrosetti’s “mild/moderate” diverticulitis stage confirmed within 24 h by CT were included. Patients with previous diverticulitis, higher Hinchey stages, or “severe” diverticulitis on Ambrosetti’s classification were excluded. The antibiotic treatment was a 10-day course of IV amoxicillin-clavulanic acid, 1200 mg four times daily for at least 48 h. After 48 h, the administration route could be switched to *per os*, 625 mg three times daily. For observational treatment, patients had to meet the criteria of tolerating a normal diet, temperature less than 100.4 °F, a pain score below 4 on a visual analogue scale (using only acetaminophen for pain control), and the ability to support self at the same level as before illness. If the patient deteriorated, CT was repeated and antibiotic treatment was started if the temperature rose above 102.2 °F, blood cultures were positive, or the patient was septic.

No significant differences between the observation and antibiotic treatment groups were found for secondary endpoints: complicated diverticulitis (3.8% vs. 2.6%, respectively; *p =* 0.377), ongoing diverticulitis (7.3% vs. 4.1%, respectively; *p =* 0.183), recurrent diverticulitis (3.4% vs. 3.0%, respectively; *p =* 0.494), sigmoid resection (3.8% vs. 2.3%, respectively; *p =* 0.323), readmission (17.6% vs. 12.0%, respectively; *p =* 0.148), adverse events (48.5% vs. 54.5%, respectively; *p =* 0.221), and mortality (1.1% vs. 0.4%, respectively; *p =* 0.432). Hospital stay was significantly shorter in the observation group (2 vs. 3 days; *p =* 0.006). However, even if no significant differences between Hinchey stages 1a and 1b diverticulitis were found, the vast majority of patients included had a diagnosis of Hinchey stage 1a ALCD (90.1% in the observational and 94% in the antibiotic-treated group) with only a small percentage of patients with Hinchey stage 1 stage 1b diverticulitis. Based on these results, the authors concluded that antibiotics can be safely omitted in patients with a first episode of uncomplicated (Hinchey 1a) ALCD. Similar results were found for Hinchey 1b diverticulitis. However, since the trial lacked power to detect smaller subgroup effects and there are no other reports in literature, the authors concluded that observational treatment should be limited to Hinchey 1a cases [44].

More recently, the long-term effects of omitting antibiotics in uncomplicated ALCD were assessed after 24 months’ follow-up of the DIABOLO trial [44]. Complete case analyses showed no difference in rates of recurrent diverticulitis (15.4% in the observational group vs. 14.9% in the antibiotic group; *p* = 0.885), complicated diverticulitis (4.8% vs. 3.3%; *p* = 0.403), and sigmoid resection (9.0% vs. 5.0%; *p* = 0.085). Young patients (< 50 years) and patients with a pain score at presentation of 8 or higher on a visual analogue pain scale were at risk for complicated or recurrent diverticulitis. In this multivariable analysis, treatment type (with or without antibiotics) was not an independent predictor for complicated or recurrent diverticulitis [45].

Although the above studies have shown there is limited evidence that antibiotics should be routinely administered to patients with uncomplicated diverticulitis, it is understood that disease severity varies in uncomplicated diverticulitis and that further studies are needed to better risk-stratify these patients in order to determine the appropriate treatment course.

The high mortality associated with sepsis requires clinicians to maintain a high index of clinical suspicion, in the conditions that predispose to sepsis in high-risk patients [46]. The expert panel suggests antibiotic therapy covering Gram-negative bacilli and anaerobes in patients with radiological documented uncomplicated acute diverticulitis associated with systemic manifestations of infection or in high-risk patients such as immunocompromised patients, elderly patients, and those with comorbidities.

If antibiotic therapy is necessary, oral administration of antibiotics may be equally as effective as intravenous administration. An expeditious switch from intravenous to oral may allow a rapid patient discharge.

A randomized controlled trial (RCT) of oral versus intravenous therapy for clinically diagnosed acute uncomplicated diverticulitis was published in 2009 [47]. Oral and intravenous regimens utilizing ciprofloxacin and metronidazole were compared. There were 41 patients in the oral arm and 38 in the IV arm (*n* = 79). No patients had to be converted to intravenous antibiotics from the oral group. There was a complete resolution of symptoms in both groups. No studies have examined the value of dietary restriction or bed rest [48].

## Could patients with uncomplicated ALCD be treated as outpatient?

*We suggest management in an outpatient setting for patients with uncomplicated ALCD and no comorbidities. We suggest re-evaluation within 7 days. If the clinical condition deteriorates, re-evaluation should be carried out earlier (weak recommendation based on moderate-quality evidence, 2B).*

Patients with uncomplicated acute diverticulitis symptoms without significant comorbidities, who are able to take fluids orally and manage themselves at home, can be treated as outpatients. They should be re-evaluated within 7 days from the time of the diagnosis. However, if the clinical condition deteriorates, re-evaluation should be carried out earlier. Patients with significant comorbidities and unable to take fluids orally should be treated in hospital with intravenous fluids.

Etzioni et al. [49] in 2010 published a retrospective analysis, demonstrating that outpatient treatment was effective for the vast majority (94%) of patients suffering from acute diverticulitis. A systematic review on outpatient management of acute uncomplicated acute diverticulitis was recently published [50]. Jackson et al. concluded that current evidence suggested that a more progressive, ambulatory-based approach to the majority of cases of acute uncomplicated acute diverticulitis was justified. Rodríguez-Cerrillo et al. [51] have recently shown that also elderly patients with comorbidities can be safely treated at home avoiding hospital admission.

The DIVER trial [52] has demonstrated that outpatient treatment may be safe and effective in selected patients with uncomplicated acute diverticulitis and can reduce the costs without negatively influencing the quality of life of these patients. This multicenter, RCT included patients older than 18 years with acute uncomplicated diverticulitis. All the patients underwent abdominal CT. The first dose of antibiotic was given intravenously to all patients in the emergency department, and then, patients were either admitted to hospital or discharged. Among a total of 132 patients, four patients in those admitted to hospital and three patients in those discharged to home management developed treatment failure (there were no differences between the groups (*p =* 0.62)). The overall health care cost per episode was 3 times less in the outpatient treated group, with significant costs savings of €1124.70 per patient. No differences were observed between the groups in terms of quality of life.

A systematic review including 21 studies (11 prospective, 9 retrospective, and only 1 randomized trial) with 1781 patients who had outpatient management of ALCD was recently published [53]. The meta-analysis concluded that outpatient management is safe, and the overall failure rate in an outpatient setting was 4.3% (95% CI 2.6–6.3%). Location of diverticulitis is not a selection criterion for an outpatient strategy (*p* = 0.512). The other subgroup analyses did not report any factors that influence the rate of failure: previous episodes of acute diverticulitis (*p* = 0.163), comorbidities (*p* = 0.187), pericolic gas (*p* = 0.653), intra-abdominal abscess (*p* = 0.326), treatment according to a registered protocol (*p* = 0.078), type of follow-up (*p* = 0.700), type of antibiotic treatment (*p* = 0.647), or diabetes (*p* = 0.610). In patients who failed outpatient treatment, the majority had prolonged antibiotic therapy and only few had percutaneous drainage for an abscess (0.13%) or surgical intervention for perforation (0.06%). However, these results should be interpreted with some caution because of the low quality of available data. The data reported suggested that outpatient management is safe if associated with an accurate selection of patients (40%); no subgroup analysis demonstrated significant differences between groups (including comorbidities, previous episode and diabetes). The main limitations of the findings of the present review concern their applicability in common clinical practice as it was impossible to identify strict criteria of failure.

Another review about outpatient management of ALCD was published in 2017 [54]. The search yielded 192 publications. Of these, 10 studies met the inclusion criteria including 1 RCT, 6 clinical controlled trials, and 3 case series. There was no difference in failure rates of medical treatment (6.5 vs. 4.6%, *p =* 0.32) or in recurrence rates (13.0 vs. 12.1%, *p =* 0.81) between those receiving ambulatory care and inpatient care for uncomplicated diverticulitis. Ambulatory treatment was associated with an estimated daily cost savings of between €600 and €1,900 per patient treated. Meta-analysis of data was not possible due to heterogeneity in study designs and inclusion criteria.

## What is the best treatment for patients with acute diverticulitis with CT findings of pericolic gas?

*In patients with CT findings of pericolic extraluminal gas, we suggest a trial of non-operative treatment with antibiotic therapy (weak recommendation based on low-quality evidence, 2C).*

High mortality associated with sepsis requires maintaining a high index of clinical suspicion for deterioration and more aggressive management. WSES expert panel recommends antibiotic therapy in patients with pericolic extraluminal gas [27]. A sub-analysis of the DIABOLO trial was recently published [55]. All patients with Hinchey 1a diverticulitis and with isolated pericolic gas on CT were identified. Pericolic gas was defined as gas located < 5 cm from the affected segment of colon. The primary outcome of the study was failure of non-operative management that was defined as need for percutaneous abscess drainage or emergency surgery within 30 days after presentation. A multivariate logistic regression analyses of clinical, radiological, and laboratorial parameters with respect to treatment failure was performed. A total of 109 patients were included. Fifty-two (48%) patients were treated with antibiotics. Nine (8%) patients failed non-operative management, seven (13%) in the antibiotic treatment group and two (4%) in the non-antibiotic group (*p* = 0.083). Only increased CRP level at presentation was an independent predictor for treatment failure. The authors concluded that non-operative treatment in diverticulitis patients with isolated pericolic gas is a suitable treatment strategy. However, due to the low event rate, it remains uncertain whether antibiotic treatment is necessary in patients with isolated pericolic gas.

## What is the best treatment for patients with a small diverticular abscess (< 4–5 cm)? What is the best treatment for patients with large diverticular abscess?

*For patients with a small (< 4–5 cm) diverticular abscess, we suggest an initial trial of non-operative treatment with antibiotics alone (weak recommendation based on low-quality evidence, 2C).*

*We suggest to treat patients with large abscesses with percutaneous drainage combined with antibiotic treatment; whenever percutaneous drainage of the abscess is not feasible or not available, we suggest to initially treat patients with large abscesses with antibiotic therapy alone, clinical conditions permitting. Alternatively, an operative intervention is required (weak recommendation based on low-quality evidence, 2C).*

Approximately 15–20*%* of patients admitted with acute diverticulitis have an abscess on CT scan [56]. The treatment of abscess always requires antibiotic therapy. If the abscess is limited in size, systemic antibiotic therapy alone is considered safe and effective in removing the abscess and solving acute inflammation with a pooled failure rate of 20% and a mortality rate of 0.6% [57].

When abscess diameter is larger, antibiotics could fail to reach the adequate concentration inside the abscess leading to an increased failure rate.

The size of 4–5 cm may be a reasonable limit between antibiotic treatment alone, versus percutaneous drainage combined with antibiotic treatment in the management of diverticular abscesses [58–62]. When the patient’s clinical conditions allow it and percutaneous drainage is not feasible, antibiotic therapy alone can be considered. However, careful clinical monitoring is mandatory. A high suspicion for surgical control of the septic source should be maintained and a surgical treatment should be performed if the patient shows a worsening of inflammatory signs or the abscess does not reduce with medical therapy.

There are currently no randomized studies available on the best treatment of intra-abdominal abscess from acute diverticulitis, and current recommendations are based only on observational studies. A retrospective study comparing outcomes of selected patients treated with initial antibiotics alone versus percutaneous drainage was published in 2015 by Elagili et al. [63]. All patients with diverticular abscess ≥ 3 cm in diameter treated in a single institution in 1994–2012 with percutaneous drainage or antibiotics alone followed by surgery were identified from an institutional diverticular disease database. Groups were compared based on patient and disease characteristics, treatment failures, and postoperative outcomes. Thirty-two patients were treated with antibiotics alone because of either technically impossible percutaneous drainage or surgeon preference, while 114 underwent percutaneous drainage. Urgent surgery was required in 8 patients with persistent symptoms during treatment with antibiotics alone (25*%*) and in 21 patients (18*%*) after initial percutaneous drainage (*p =* 0.21). Patients treated with antibiotics had a significantly smaller abscess diameter (5.9 vs. 7.1 cm, *p =* 0.001) and shorter interval from initial treatment to sigmoidectomy (mean 50 vs. 80 days, *p =* 0.02). Postoperative complications following antibiotics alone were significantly less severe than after percutaneous drainage based on the Clavien-Dindo classification (*p =* 0.04).

In patients displaying an appropriate clinical improvement, the drainage catheter can be removed when the output has ceased or decreased substantially. In doubtful cases, a CT scan can be performed with water-soluble contrast via the percutaneous drainage catheter prior to drain removal. If no identifiable cavity remains, the catheter should be removed. If resolution of the abscess is not reached and the patient has no clinical improvement, further drainage or catheter repositioning might be indicated and may eventually necessitate surgery.

## Should an early colonic evaluation be planned in patients treated non-operatively for a diverticular abscess? Should an early colonic evaluation be recommended for patients with a CT-proved uncomplicated acute diverticulitis treated non-operatively?

*In patients with diverticular abscesses treated non-operatively, we suggest to plan an early colonic evaluation (4–6 weeks) (weak recommendation based on low-quality evidence, 2C).*

*In patients with CT-proven uncomplicated diverticulitis treated non-operatively, we do not recommend routine colonic evaluation (weak recommendation based on moderate-quality evidence, 2B).*

Colonic localized abscess is an uncommon, but possible, presentation of an occult colon malignancy, and it may mimic complicated diverticular disease [64, 65]. It has been demonstrated that the risk of malignancy after a CT-proven uncomplicated diverticulitis is low and, in the absence of other indications, routine colonoscopy may not be necessary. A systematic review investigating the rate of colorectal cancer (CRC) found by colonoscopy after an episode of uncomplicated diverticulitis was published in 2014 [66]. Nine studies met the inclusion criteria and included a total number of 2490 patients with uncomplicated diverticulitis. Subsequent colonoscopy after an episode of uncomplicated diverticulitis was performed in 1468 patients (59%). Seventeen patients were diagnosed with CRC, having a prevalence of 1.16% (95% confidence interval 0.72–1.9% for CRC). Hyperplastic polyps were seen in 156 patients (10.6%), low-grade adenoma in 90 patients (6.1%), and advanced adenoma in 32 patients (2.2%). The results of this review demonstrate that unless colonoscopy is regarded for screening in individuals aged 50 years and older, routine colonoscopy in the absence of other clinical signs of CRC is not required in patients following an episode of acute uncomplicated diverticulitis.

Another systematic review and meta-analysis on the role of routine colonic evaluation after radiologically confirmed acute diverticulitis was published in 2014 [67]. Eleven studies from 7 countries were included in the analysis. Among 1970 patients, cancer was only found in 22 (0.01%) cases. The risk of malignancy after a radiologically proven episode of acute uncomplicated diverticulitis was low. Patients with complicated diverticulitis had a significant risk of CRC at subsequent colonic evaluation.

A retrospective study of 633 patients with acute diverticulitis diagnosed by CT was published in 2014 [68]. Of the 663 patients, 97 patients underwent emergency resection, while 536 patients were treated non-operatively, 394 of whom subsequently underwent colonoscopy. The findings showed 17 cancers (2.7%) in patients with an initial diagnosis of acute diverticulitis. As shown by CT, 16 cancer patients (94*%*) had an abscess, while one patient had pericolic extraluminal gas but no abscess. Of the patients with an abscess, 11.4% had cancer mimicking acute diverticulitis. No cancer was found in the patients with uncomplicated diverticulitis.

## What is the role of non-operative treatment in patients with CT findings of distant gas without diffuse intra-abdominal fluid?

*In patients with CT findings of distant free gas without diffuse intra-abdominal fluid, we suggest a non-operative treatment in selected patients only if a close follow-up can be performed (weak recommendation based on very low-quality evidence, 2D).*

Although most patients hospitalized for acute diverticulitis can be managed by non-operative treatment, up to 25% may require urgent operative intervention [69]. Patients with diffuse peritonitis are typically critically ill patients and require prompt fluid resuscitation, antibiotic administration, and surgery. While the absolute prevalence of perforated diverticulitis complicated by generalized peritonitis is low, it is associated with significant postoperative mortality, regardless of selected surgical strategy.

Despite CT findings of distant free gas (a known predictor of failure of non-operative treatment [27]), Dharmarajan et al. [70] described a high success rate for non-operative management in patients with acute diverticulitis and a pneumoperitoneum, excluding those with hemodynamic instability. Sallinen et al. [71] reported results of non-operative management in patients with CT verified extraluminal gas. The study showed that non-operative treatment was feasible therapy only for hemodynamically stable patients with pericolic extraluminal gas or with small amount of distant intraperitoneal gas in the absence of clinical diffuse peritonitis or fluid in the fossa Douglas. Occurrence of large amount of distant intraperitoneal gas or distant retroperitoneal gas even in the absence of clinical generalized peritonitis was associated with high failure rate (57–60%) of non-operative management. Moreover, nearly 60% patients with distant intraperitoneal gas were primarily treated by surgery.

Highly selected group of patients at this stage may be treated by conservative treatment. However, it may be associated with a significant failure rate and a careful clinical and CT monitoring is mandatory [20]. Suggested intervention for patients at this stage should be surgical resection and anastomosis with or without stoma in stable patients without comorbidities, and Hartmann’s procedure (HP) in unstable patients or in patients with multiple comorbidities [27].

## Should laparoscopic lavage and drainage be recommended in patients with diffuse peritonitis due to diverticular perforation?

*We suggest performing laparoscopic peritoneal lavage and drainage only in very selected patients with generalized peritonitis. It is not considered as the first line treatment in patients with peritonitis from acute colonic diverticulitis (weak recommendation based on high-quality evidence, 2A).*

A minimally invasive approach using laparoscopic peritoneal lavage and drainage has been debated in recent years as an alternative to colonic resection [72]. It can potentially avoid a stoma in patients with diffuse peritonitis. It consists of the laparoscopic aspiration of pus followed by abdominal lavage and the placement of abdominal drains, which remain for many days after the procedure. In 2013, a Dutch retrospective analysis of 38 patients [73] treated by laparoscopic lavage was published highlighting some doubts about this procedure to treat critically ill patients. In seven patients, this approach did not control abdominal sepsis, two patients died of multiple organ failure and five ones required further surgical interventions (three Hartmann resection, one diverting stoma, and one perforation closure). One of these died from aspiration, and the remaining four experienced prolonged and complicated hospital stay. Multiple comorbidities, IMS, a high CRP level, and/or a high Mannheim Peritonitis Index were also predictors of a high risk of failure. The authors concluded that patient selection was of utmost importance and identification of an overt sigmoid perforation is of critical importance. Great debate is still open on this topic, mainly due to the discrepancy and sometime disappointing results of the latest prospective trials such as SCANDIV, Ladies, and DILALA trials [74–76]

In 2014, the first results from the RCT DILALA were published [74]. Initial diagnostic laparoscopy showing Hinchey III disease was followed by randomization between laparoscopic lavage and colon resection and stoma. Morbidity and mortality after laparoscopic lavage did not differ when compared with the Hartmann procedure. Laparoscopic lavage resulted in shorter operating time, shorter time in the recovery unit, and shorter hospital stay with the avoidance of a stoma. In this trial, laparoscopic lavage as treatment for patients with perforated diverticulitis Hinchey III disease was feasible and safe in the short-term. In 2015, the results of SCANDIV study were published [75]. Among patients with likely perforated diverticulitis and undergoing emergency surgery, the use of laparoscopic lavage vs. primary resection did not reduce severe postoperative complications and led to worse outcomes in secondary endpoints. These findings do not support laparoscopic lavage for treatment of perforated diverticulitis. In the same year, the result of LADIES study was published. This showed that laparoscopic lavage was not superior to sigmoidectomy for the treatment of purulent perforated diverticulitis [76].

After their publication, the results of the three studies were summarized in six different meta-analyses, with similar findings [77–82]. When compared with emergency surgery with resection, laparoscopic lavage in Hinchey III acute diverticulitis shows a comparable mortality but is associated with a failure rate with a significantly augmented need for reoperation due to the failure of the treatment and to intra-abdominal abscess formation. Long-term results were similar, with no difference in morbidity and mortality.

Several controversies remain about laparoscopic lavage and drainage. It may be an acceptable alternative in selected patients [83]; however, it cannot be considered the first line treatment in patients with diverticular peritonitis.

## Should primary anastomosis with or without protecting stoma be preferred instead of Hartmann’s procedure in patients with diffuse peritonitis from diverticular perforation?

*We recommend Hartmann’s procedure (HP) for managing diffuse peritonitis in critically ill patients and in selected patients with multiple comorbidities (strong recommendation based on low-quality evidence, 2B).*

*In clinically stable patients with no comorbidities, we suggest primary resection with anastomosis with or without a diverting stoma (weak recommendation based on low-quality evidence, 2B).*

HP has been considered the procedure of choice in patients with generalized peritonitis and remains a safe technique for emergency colectomy in diverticular peritonitis, and is especially useful in critically ill patients and in patients with multiple comorbidities. However, restoration of bowel continuity after a HP is associated with significant morbidity and resource utilization [84]. As a result, many of these patients do not undergo reversal surgery and remain with a permanent stoma [85].

Common use of the HP in treating diverticular perforation worldwide is confirmed by a recent Australian study analyzing administrative data of patients with acute diverticulitis admitted, from 2009 to 2013, in eight tertiary referral centers with specialist colorectal services [86]. The HP was the most commonly performed emergency operation, accounting for 72% of resections.

Another population-based retrospective cohort study using administrative discharge data, conducted in Ontario, Canada, was published in 2014 [87]. Among 18,543 patients hospitalized with a first episode of diverticulitis, from 2002 to 2012, 3873 underwent emergency surgery. The use of laparoscopy increased (9 to 18%, *p* < 0.001), whereas the use of the HP remained unchanged (64%), and like in the Australian study, was the most frequently used operative approach in patients with complicated acute diverticulitis.

In recent years, some authors have reported the role of primary resection and anastomosis with or without a diverting stoma, in the treatment of acute diverticulitis, even in the presence of diffuse peritonitis [88]. The decision regarding the surgical choice in patients with diffuse peritonitis is generally left to the judgment of the surgeon, who takes into account the clinical condition and the comorbidities of the patient. Studies comparing mortality and morbidity of the HP versus primary anastomosis did not show any significant differences. However, most studies had relevant selection biases, as demonstrated by four systematic reviews [89–91].

A study evaluating all patients with acute diverticulitis undergoing emergent primary anastomosis with diverting loop ileostomy and HP was recently published using the ACS-NSQIP Colectomy Procedure Targeted Database from 2012 to 2016 [92]. Out of 130,963 patients, 2729 patients were included. The median age was 64 years, and 48.5% were male; the majority of patients underwent a HP, and only 208 (7.6%) underwent primary anastomosis with diverting loop ileostomy. Patients undergoing a HP had more comorbidities [e.g., COPD (9.8% vs. 4.8%, *p =* 0.017)], were more functionally dependent [6.3% vs. 2.4%, *p =* 0.025], and were more unwell [e.g., septic shock (11.1% vs. 5.3%, *p =* 0.015)] compared to primary anastomosis with diverting loop ileostomy patients. The mortality rates for the patients undergoing a HP versus primary anastomosis with diverting loop ileostomy were 7.6% and 2.9%, respectively (*p =* 0.011). The morbidity rates were 55.4% and 48.6%, respectively (*p =* 0.056). In multivariable analyses, compared to the HP, primary anastomosis with diverting loop ileostomy did not result in increased rates of mortality (OR = 0.21, 95% CI 0.03–1.58, *p =* 0.129) or morbidity (OR = 0.96, 95% CI 0.63–1.45, *p =* 0.834). The authors concluded that primary anastomosis with diverting loop ileostomy appears to be at least a safe alternative to the HP for select patient populations needing emergent surgical management of acute diverticulitis.

A comparison of primary resection and anastomosis with or without defunctioning stoma to the HP as the optimal operative strategy for patients presenting with Hinchey stage III–IV was published by Constantinides et al. [93]. A total of 135 primary resection and anastomosis, 126 primary anastomosis with defunctioning stoma, and 6619 HPs were considered in the study. Morbidity and mortality were 55% and 30% for primary resection and anastomosis, 40% and 25% for primary anastomosis with defunctioning stoma, and 35% and 20% for the HP. Stomas remained permanent in 27% of HPs and in 8% of primary anastomoses with defunctioning stoma. The authors concluded that primary anastomosis with defunctioning stoma may be the optimal strategy for selected patients with diverticular peritonitis and may represent a good compromise between postoperative adverse events, long-term quality of life, and risk of permanent stoma.

A small randomized trial of primary anastomosis with ileostomy versus a HP in patients with diffuse diverticular peritonitis was published by Oberkofler et al. in 2012 [94]. Sixty-two patients with acute left-sided colonic perforation (Hinchey III and IV) from 4 centers were randomized to Hartmann procedure (*n* = 30) and to primary anastomoses with diverting ileostomy (*n* = 32). A planned stoma reversal operation was performed after 3 months in both groups. The study reported no difference in initial mortality and morbidity (mortality 13% vs. 9% and morbidity 67% vs. 75% in the HP vs. primary anastomosis), but a reduction in length of stay, lower costs, fewer serious complications, and greater stoma reversal rates in the primary anastomosis group.

A multicenter RCT conducted between June 2008 and May 2012, the DIVERTI (Primary vs. Secondary Anastomosis for Hinchey Stage III-IV Diverticulitis) trial [95], was published in 2017. All 102 patients enrolled were comparable for age (*p =* 0.4453), sex (*p =* 0.2347), Hinchey stage III vs. IV (*p =* 0.2347), and Mannheim Peritonitis Index (*p =* 0.0606). Overall mortality did not differ significantly between the HP (7.7%) and primary anastomosis (4%) (*p =* 0.4233) groups. Morbidity for both resection and stoma reversal operations was comparable (39% in the HP arm vs. 44% in the primary anastomosis arm; *p =* 0.4233). At 18 months, 96% of primary anastomosis patients and 65% of the HP patients had a stoma reversal (*p =* 0.0001). Although mortality was similar in both arms, the rate of stoma reversal was significantly higher in the primary anastomosis arm. This trial provides additional evidence in favor of primary anastomosis with diverting ileostomy over the HP in patients with diverticular peritonitis.

In 2019, the results of the LADIES study [96] demonstrated that in hemodynamically stable, immunocompetent patients younger than 85 years, primary anastomosis is preferable to the HP as a treatment for perforated diverticulitis (Hinchey III or Hinchey IV disease). Patients aged between 18 and 85 years who presented with clinical signs of general peritonitis and suspected perforated diverticulitis were eligible for inclusion if plain abdominal radiography or CT scan showed diffuse free gas or fluid. Patients with Hinchey I or II diverticulitis were not eligible for inclusion. Patients were allocated (1:1) to the HP or sigmoidectomy with primary anastomosis, with or without defunctioning ileostomy. The 12-month stoma-free survival was significantly better for patients undergoing primary anastomosis compared with the HP (94.6% [95% CI 88.7–100] vs. 71.7% [95% CI 60.1–83·3], hazard ratio 2.79 [95% CI 1·86–4.18]; log-rank *p <* 0·0001). There were no significant differences in short-term morbidity and mortality after the index procedure for the HP compared with primary anastomosis (morbidity, 29 [44%] of 66 patients vs. 25 [39%] of 64, *p =* 0.60; mortality, two [3%] vs. four [6%], *p =* 0.44).

Recently, a systematic review of the existing literature about surgical management of Hinchey III and IV diverticulitis was published [97]. A total of 25 studies involving 3546 patients were included in this study. The overall mortality in patients undergoing a HP was 10.8% across the observational studies and 9.4% in the RCTs. The mortality rate in patients undergoing a primary anastomosis was lower than that in the HP group, at 8.2% in the observational studies and 4.3% in the RCTs. A comparison of primary anastomosis with the HP demonstrated a 40% lower mortality rate in the primary anastomosis group than in the HP group (OR 0.60, 95% CI 0.38–0.95, *p =* 0.03), when analyzing the observational studies. However, meta-analysis of the RCTs did not demonstrate any difference in mortality. Wound infection rates between the two groups were comparable.

## Should laparoscopic resection be preferred to open resection in patients with diffuse peritonitis due to perforated diverticulitis?

*In patients with diffuse peritonitis due to perforated diverticulitis, we suggest to perform an emergency laparoscopic sigmoidectomy only if technical skills and equipment are available (weak recommendation based on low-quality evidence, 2C).*

Laparoscopic sigmoidectomy for diverticulitis had initially been confined to the elective setting. However, in physiologically stable patients, laparoscopic sigmoidectomy may be feasible in the setting of purulent and fecal diverticular peritonitis. In 2015, a systematic review on laparoscopic sigmoidectomy for diverticulitis in the emergency setting was published [98].

The review included 4 case series and one cohort study (total of 104 patients) out of 1706 references. A HP was performed in 84 patients, and primary anastomosis was fashioned in 20 patients. The mean operating time varied between 115 and 200 min. The conversion to open surgery rate varied between 0 and 19%. The mean length of hospital stay ranged between 6 and 16 days. Surgical re-intervention was necessary in 2 patients. In 20 patients operated upon without defunctioning ileostomy, no anastomotic leakage was reported. Three patients died during the postoperative period. Stoma reversal after HP was performed in 60 out of 79 evaluable patients (76%).

These guidelines are limited by the low-quality evidence that showed that emergency laparoscopic sigmoidectomy for the treatment of perforated diverticulitis with generalized peritonitis is feasible. These studies occurred in selected patients and in experienced units and are not generalizable to all centers. High-quality prospective or randomized studies are needed to demonstrate benefits of emergency laparoscopic sigmoidectomy compared to open sigmoidectomy for perforated diverticulitis.

## Should damage control surgery with staged laparotomies be recommended in patients with acute peritonitis due to diverticular perforation?

*We suggest damage control surgery (DCS) with staged laparotomies in selected unstable patients with diffuse peritonitis due to diverticular perforation (weak recommendation based on low-quality evidence, 2C).*

A damage control surgical strategy may be useful for patients in physiological extremis from abdominal sepsis [99]. The initial surgery focuses on control of the sepsis, and a subsequent operation deals with the anatomical restoration of the gastrointestinal tract, after a period of physiological resuscitation. This strategy facilitates both the control of the severe sepsis control as well as potentially improving the rate of primary anastomosis [100].

Generalized diverticular peritonitis is a life-threatening condition requiring prompt emergency operation. To improve outcomes and reduce the rate of colostomy formation, a new algorithm for damage control operation, lavage, limited resection or closure of perforation, and second look surgery to restore intestinal continuity was developed in recent years [101, 102]. Some patients may be physiologically deranged. These patients, who are hemodynamically unstable, are not optimal candidates for immediate complex operative interventions. After initial surgery, which should be limited to source control, e.g., primary closure of the perforation/local resection of the diseased bowel, the patient is taken to the intensive care unit (ICU) for physiologic optimization. However, this strategy will also delay bowel anastomosis to a period of physiological stability [103] potentially changing the intraoperative physiological milieu, potentially favoring a primary anastomosis, and avoiding the formation of a stoma altogether. In the setting of acute diverticulitis, several reports (with low level of evidence) were published. In 2010, a prospective observational study was published by Kafka-Ritsch et al. [101]. A total of 51 patients (28 females 55%) with a median age of 69 (range 28–87) years, with perforated diverticulitis Hinchey III (*n* = 40, 78%) or Hinchey IV (*n* = 11, 22%), were prospectively enrolled in the study. Patients were initially managed with limited resection, lavage, and temporary abdominal closure followed by second, reconstructive operation 24–48 h later, which are supervised by a colorectal surgeon. Bowel continuity was restored in 38 (84%) patients, of which four were protected by a loop ileostomy. Five anastomotic leaks (13%) were encountered requiring loop ileostomy in two patients and a HP in remaining three patients. The overall mortality rate was 9.8%, and 35 of 46 surviving patients (76%) left the hospital with reconstructed colon continuity. Fascial closure was achieved in all patients.

Sohn et al. performed a case-control study comparing traditional strategy versus damage control: there were no differences in morbidity and mortality, but there was a significant reduction of stoma creation in the damage control group [104].

Despite promising experiences, little robust or large-scale data are available, and the open abdomen and damage control strategy are not without risk: for example, such procedures are associated with the formation of entero-atmospheric fistula and high costs, among other issues. Guidelines recommend this strategy only in critically ill patients who cannot withstand major surgery. Although there is now a biologic rationale for such an intervention as well as non-standardized and erratic clinical utilization, this remains a novel therapy with potential side effects and clinical equipoise. The WSES recommends to use an open abdomen approach in selected significantly physiologically deranged patients with ongoing sepsis [105]. The Closed Or Open after Laparotomy (COOL) study constitutes a prospective RCT that will randomly allocate eligible surgical patients intraoperatively to either formal closure of the fascia or use of the open abdomen with application of with active negative peritoneal pressure therapy. This trial will be powered to demonstrate a mortality difference in this highly lethal and morbid condition to ensure critically ill patients are receiving the best care possible and not being harmed by inappropriate therapies based on opinion only [106].

## What factors should be considered in planning elective resection in cases of acute diverticulitis treated non-operatively?

*We suggest evaluating patient-related factors and not number of previous episodes of diverticulitis in planning elective sigmoid resection (weak recommendation based on very low-quality evidence, 2D).*

*After an episode of ALCD treated conservatively, we suggest planning of an elective sigmoid resection in high-risk patients, such as immunocompromised patients (weak recommendation based on very low-quality evidence, 2D).*

Recurrence of acute diverticulitis is lower than previously thought. Historically, it has been reported that about one third of all patients with acute diverticulitis will have a recurrent attack, often within 1 year [107, 108]. However, the recurrence after an uncomplicated episode of diverticulitis appears much lower: with a recent prospective study reported a recurrence of only 1.7% over 5 years of follow-up [109, 110]. After a follow-up of 4 years, El Sayed et al. [111], in an English study of over 65,000 patients managed non-operatively for their first episode of diverticulitis, found the recurrence rate to be around 11.2%. Emergency and elective colectomy rates were 0.9 and 0.75%, respectively. Female gender, young age, smoking, obesity, and complicated initial disease were risk factors for readmission and emergency surgery. The study also pointed out that some factors associated with recurrence are modifiable; weight reduction and smoking cessation can be championed.

In 2014, a systematic review of studies reviewing the diagnosis and management of chronic and recurrent diverticulitis (from studies published between January 2000 to March 2013) was published [112]. The 68 studies included were almost exclusively observational and had limited certainty of treatment effect. The authors found that complicated recurrence after recovery from an uncomplicated episode of diverticulitis was rare (< 5%) and that age at onset younger than 50 years and 2 or more recurrences did not increase the risk of complications.

The authors concluded that the indication for elective colectomy following 2 episodes of diverticulitis is no longer accepted. Indication to colectomy should be made based on consideration of the risks of recurrent diverticulitis, the morbidity of surgery, ongoing symptoms, the complexity of disease, and operative risk.

A recent open-label randomized multicenter trial (DIRECT trial) randomized 109 patients from 24 teaching and two academic hospitals in the Netherlands presenting with recurrent and persisting abdominal complaints after an episode of diverticulitis to receive surgical treatment or non-operative management [113]. After a brief follow-up of 6 months, elective sigmoidectomy resulted in a better quality of life (assessed by many specific questionnaires) compared to non-operative management. However, the results of the study may be affected by the heterogeneity of patients enrolled (patients with both recurrent diverticulitis and patients with persistent abdominal complaints).

Currently, the decision to perform an elective resection after one or more episodes of AD should be undertaken on a case-by-case basis, taking into account risk factors, complications, age, and severity of episodes as well as the patient’s personal circumstances and comorbidities (e.g., immunosuppressed patients) [114].

## What is the optimal antibiotic therapy for patients with diffuse peritonitis due to diverticular perforation? What is the optimal duration of antibiotic therapy after surgical source control in diffuse peritonitis due to diverticular perforation?

*We suggest to choose the empirically designed antibiotic regimen on the basis of the underlying clinical condition of the patient, the pathogens presumed to be involved, and the risk factors for major antimicrobial resistance patterns (strong recommendation based on moderate-quality evidence, 1B).*

*We suggest a 4-day period of postoperative antibiotic therapy in complicated ALCD if source control has been adequate (weak recommendation based on moderate-quality evidence, 2B).*

Antibiotic therapy plays an important role in the management of complicated acute diverticulitis. Typically, it is an empiric antibiotic treatment. The regimen should depend on the severity of infection, the pathogens presumed to be involved, and the risk factors indicative of major resistance patterns [39]. Several recommendations have been recently published in literature [39]. However, consideration of local epidemiological data and resistance profiles is essential for antibiotic selection.

Considering intestinal microbiota of large bowel acute diverticulitis requires antibiotic coverage for Gram-positive and Gram-negative bacteria, as well as for anaerobes. Most of the complicated acute diverticulitis is mainly a community-acquired infection. The main resistance threat in IAIs is posed by extended-spectrum beta-lactamase (ESBL)-producing *Enterobacteriaceae*, which are becoming increasingly common in community-acquired infections worldwide [33]. The most significant risk factors for ESBL-producing pathogens include prior exposure to antibiotics and comorbidities requiring concurrent antibiotic therapy [39]. Anti-ESBL-producer coverage should be warranted for patients with these risk factors. Discontinuation of antibiotic treatment should be at 4 days from source control as this has been demonstrated as non-inferior to longer therapy based on the STOP IT trial [115].

The recent prospective trial by Sawyer et al. demonstrated that in patients with complicated IAIs undergoing an adequate source-control procedure, the outcomes after approximately 4 days fixed-duration antibiotic therapy were similar to those after a longer course of antibiotics that extended until after the resolution of physiological abnormalities [115].Patients who have signs of sepsis beyond 5 to 7 days of adequate antibiotic treatment warrant aggressive diagnostic investigation in search of a reservoir of infection.

## Which are the principles of the treatment of acute right-sided colonic diverticulitis?

*Although studies have shown that the percentage of complications requiring surgery is higher in patients with ALCD than in patients with ARCD, the principles of diagnosis and treatment of patients with ARCD are similar to those with ALCD. We suggest that all the statements for ALCD also apply to ARCD.*

Acute colonic diverticulitis is a common condition affecting the adult population. Traditionally, the sigmoid colon is considered the most commonly involved part, and ARCD is much rarer [116]. However, in some regions of the world, ARCD outnumber ALCD [116]. The ARCD differs from the ALCD in some aspects. The former is usually solitary [29, 117], and has a low rate of complicated diverticulitis [118].

ARCD generally occurs in middle-aged men, and its incidence does not increase with age. Especially the ARCD located in the cecum, it is difficult to distinguish ARCD from acute appendicitis because of their similar symptoms and signs.

CT scanning appears to be the best overall imaging modality in the diagnosis of possible ARCD [119, 120]. However, US is more economic than CT and poses no radiation, which may be particularly important since the patients having right-sided diverticulitis are relatively younger.

US features, including diverticular wall thickening, surrounding echogenic fat, and intra-diverticular echogenic material, can provide clear information for making correct preoperative diagnosis. However, US is operator dependent. Ambiguous US studies may be complemented with a contrast-enhanced CT [121].

Currently, the management of ARCD is not well defined, and no unique guidelines have been proposed.

Although previous studies have shown that the percentage of complications requiring surgery is higher in patients with ALCD than in patients with ARCD [122], the principles of diagnosis and treatment of ARCD are very similar to those of ALCD. As a treatment option, non-operative methods should be preferred, in cases without diffuse peritonitis although differentiating benign and malignant cases pre-operatively is often difficult [123]. Surgical treatment is usually used in the treatment of complicated cases [116, 124, 125]. Resection of the inflamed colon with primary anastomosis can be performed by laparoscopy in experienced centers [126].

## Conclusions

ALCD is a common problem encountered by Western surgeons in the acute setting. The sigmoid is usually the most commonly involved colonic segment, while ARCD is much rarer.

An international multidisciplinary panel of experts from the World Society of Emergency Surgery (WSES) updated its guidelines on the management of acute left-sided colonic (ALCD) diverticulitis according to the most recent available literature. The update includes recent changes introduced in the management of ALCD. The new update contains a section on ARCD, which is more prevalent than ALCD in some regions of the world.

## Data Availability

Not applicable.

## Abbreviations

ALCD: Acute left-sided colonic diverticulitis
ARCD: Acute right-sided diverticulitis
CRC: Colorectal cancer
CRP: C-reactive protein
CT: Computed tomography
DCS: Damage control surgery
ESBL: Extended-spectrum beta-lactamase
HP: Hartmann’s procedure
IAIs: Intra-abdominal infections
ICU: Intensive care unit
IMS: Immunosuppression
RCT: Randomized controlled trial
US: Ultrasound
WBC: White blood cell
WSES: World Society of Emergency Surgery
